# Severity of Symptoms as an Independent Predictor of Poor Outcomes in Patients with Advanced Cancer Presenting to the Emergency Department: Secondary Analysis of a Prospective Randomized Study

**DOI:** 10.3390/cancers16233988

**Published:** 2024-11-28

**Authors:** Aiham Qdaisat, Elizabeth Stroh, Cielito Reyes-Gibby, Monica K. Wattana, Jayne Viets-Upchurch, Ziyi Li, Valda D. Page, Huda Fatima, Patrick Chaftari, Ahmed Elsayem

**Affiliations:** 1 Department of Emergency Medicine, The University of Texas MD Anderson Cancer Center, Houston, TX 77030, USA; aqdaisat@mdanderson.org (A.Q.); ellie.stroh2004@tamu.edu (E.S.); creyes@mdanderson.org (C.R.-G.); mwattana@mdanderson.org (M.K.W.); jviets@mdanderson.org (J.V.-U.); vpage@mdanderson.org (V.D.P.); hfatima@mdanderson.org (H.F.); pchaftari@mdanderson.org (P.C.); 2 Department of Biostatistics, The University of Texas MD Anderson Cancer Center, Houston, TX 77030, USA; zli16@mdanderson.org

**Keywords:** advanced cancer, symptom, delirium, emergency department, palliative care, MDASI

## Abstract

**Background/Objectives**: Patients with advanced cancer often present to the emergency department (ED) with pain and distressing symptoms that are not systematically evaluated. The current study investigated the association of symptom severity with the diagnosis of delirium and short-term survival. **Methods**: In this secondary analysis of a prospective randomized study of delirium among advanced cancer patients in the ED, in which symptoms were assessed by the MD Anderson Symptom Inventory (MDASI), we analyzed the distribution of MDASI item scores by 90-day mortality (Kolmogorov–Smirnov), the association of MDASI item scores with short-term mortality (logistic regression models), and the symptoms in those with or without delirium (Mann–Whitney U test or chi-square test). **Results**: Of the 243 patients included, 222 (91.4%) had complete MDASI scores. The MDASI median symptom scores for pain, fatigue, and interference with work were the highest. A significant difference in MDASI item score distribution with 90-day mortality was observed for fatigue (*p* = 0.018), shortness of breath (*p* < 0.001), difficulty remembering (*p* = 0.038), lack of appetite (*p* = 0.035), drowsiness (*p* < 0.001), feeling sad (*p* = 0.031), and interference with walking (*p* < 0.001). In multivariable logistic regression models, shortness of breath (adjusted OR 1.15, 95% CI 1.04–1.26, *p* = 0.005) and drowsiness (adjusted OR 1.17, 95% CI 1.05–1.33, *p* = 0.008) were associated with 90-day mortality, adjusting for age, race, performance status, and cancer type. The median total MDASI score was significantly higher in patients with delirium than in those without (88, IQR 83–118 vs. 80, IQR 55–104; *p* < 0.001). **Conclusions**: Patients with advanced cancer presenting to the ED had severe symptoms, some of which were associated with shorter survival. These findings underscore the necessity of systematic symptom assessment, focusing on shortness of breath, drowsiness, fatigue, difficulty remembering, lack of appetite, feeling sad, and feeling distressed, to enhance clinical decision-making and improve the care of patients with advanced cancer. Additional longitudinal studies are needed to evaluate the improvement in symptoms and quality of life for these patients.

## Simple Summary

Patients with advanced cancer frequently seek care in the emergency department and usually present with a constellation of symptoms. In an emergency/urgent setting, it is crucial to understand the severity of these symptoms, which include pain, fatigue, nausea, disturbed sleeping, and distress, as these can greatly influence patient outcomes and the care of cancer patients. In this study, we aimed to explore the relationship between the severity of these symptoms, the diagnosis of delirium, and short-term survival outcomes. Understanding this relationship offers important insights that aid in managing these symptoms, improving patient outcomes, and enhancing a patient’s overall quality of life.

## 1. Introduction

Patients with advanced cancer suffer from multiple severe physical and psychosocial symptoms, which can negatively impact their quality of life [1,2]. Common physical symptoms include pain, fatigue, shortness of breath, nausea and/or vomiting, constipation, and sleep disturbances, and common psychosocial symptoms include anxiety or depression. Frequently, a cluster of multiple symptoms occurs in the same patient. Furthermore, those with advanced cancer are more likely to suffer from severe symptoms at a significantly higher rate [3]. Patients often seek care in the emergency department (ED) when these symptoms become severe. Studies have shown that referral to palliative care from the ED results in the earlier control of symptoms [4,5]. However, referral to palliative care usually occurs late after admission to the hospital or in the last week of life, and aggressive end-of-life care for cancer is common [6,7].

The rate of ED visits by cancer patients has been increasing [8,9], with over 4 million visiting the ED annually, and most end up being admitted to the hospital [9]. In a multicenter study of 18 EDs, Yilmaz et al. found that 62% of patients presenting to the ED had advanced cancer; only 6.5% of these were receiving palliative care, and most were admitted to the hospital, sometimes to intensive care units [10]. Although multiple presentations to the ED at the end of life are considered an indicator of poor-quality cancer care, about 75% of cancer patients visit the ED at least once in the last 6 months of life, with 45% of them visiting the ED in the last month of life [11].

Previously, we reported that more than half of patients who visited a comprehensive cancer center had advanced cancer, defined as locally advanced or metastatic, and 18% of them had delirium, as assessed by the Memorial Delirium Assessment Scale, with a cutoff point of 7/30 [12]. Moreover, we have shown that patients with delirium had poorer performance status, worse overall survival, and higher hospitalization rates compared with patients without delirium [12,13]. Despite the presence of tools for palliative care teams to assess the severity of symptoms and quality of life in patients with advanced cancer [14,15,16], the systematic use of these tools in the ED setting is not usually reported; some studies report only the presenting symptoms.

While our previous work focused on determining the frequency and recognition of delirium among patients with advanced cancer presenting to the ED [12,13], the severity of symptoms for the enrolled patients was assessed using the MD Anderson Symptom Inventory (MDASI)—a tool that measures a broad range of cancer-related symptoms—to better understand the clinical characteristics of the patients. Though our previous findings provided valuable insights into the burden of delirium, the association between other symptoms and short-term mortality has not been reported. Recognizing the connection between symptoms and mortality risk could provide valuable information for better early risk stratification and clinical decision-making for cancer patients presenting to the emergency department. Without this critical information, identifying high-risk patients based on their symptoms becomes challenging, limiting the potential for tailored interventions that could mitigate adverse outcomes. Therefore, the current study aimed to investigate the association of symptom severity, as measured by the MDASI score, with survival outcomes.

## 2. Materials and Methods

### 2.1. Patient Cohort and Inclusion Criteria

This is a secondary analysis of a single-center prospective randomized observational study that was conducted at the University of Texas MD Anderson Cancer Center, Houston, Texas. Patients with advanced cancer presenting to our ED for care between 11 March 2013 and 21 July 2014 were enrolled. The eligibility criteria were as follows: (1) age ≥ 18 years; (2) able to provide consent or accompanied by a legally authorized representative able to provide consent; (3) able to communicate in English; and (4) in the ED for 12 h or less. Exclusion criteria were as follows: (1) unstable condition requiring emergent medical attention; (2) comatose; (3) learning disability or other communication barriers such as aphasia or deafness; and (4) documented history of dementia. Advanced cancer was defined as metastatic or locally recurrent solid cancer not amenable to curative treatment or a refractory or relapsed hematologic malignancy. Additional details of the methods used were previously reported [12]. Sample size calculations are highlighted in the supplemental data. In that study, we found a 9% frequency of delirium using the confusion assessment method and 18% using the Memorial Delirium Assessment Scale [12].

### 2.2. MD Anderson Symptom Inventory

We assessed the frequency and severity of symptoms using the MDASI [17]. This tool measures 19 items divided into two categories: 13 symptom items and 6 interference items. The symptom items assess symptoms commonly encountered by cancer patients and their severity on a scale of 0 to 10. These symptoms included pain, fatigue, nausea, vomiting, disturbed sleep, feeling distressed (upset), shortness of breath, difficulty remembering, lack of appetite, drowsiness, a dry mouth, feeling sad, and numbness or tingling. Moreover, the MDASI measures 6 interference items that include physical function (walking, working, and general activity) and psychological function (mood, relationships with other people, and enjoyment of life) [17]. Multiple prior studies have confirmed that the MDASI tool is both reliable and sensitive in evaluating symptom burden in various cancer patient groups, regardless of their cancer or treatment status [17,18,19,20,21,22]. The findings highlight strong internal consistency (the alpha value typically exceeding 0.8). Moreover, the studies emphasize its sensitivity in detecting changes in symptom severity, which supports effective patient monitoring and treatment adaptation [17,18,19,20,21,22].

### 2.3. Statistical Analysis

Descriptive statistics, including counts, percentages, means and standard deviations, or medians and interquartile ranges (IQR), were used to summarize the patient characteristics. The continuous variables’ normality was evaluated with histograms, box plots, Q-Q plots, and the Shapiro–Wilk test, for which none of the variables met the assumption of normality. Density plots were used to visualize the distribution of different MDASI item scores stratified by 90-day mortality and were compared using the Kolmogorov–Smirnov test. Univariate logistic regression analysis was performed to determine the association between each MDASI item and 90-day mortality as the primary outcome. Secondary outcomes were 14- or 30-day mortality. MDASI items have been shown to be highly correlated. To account for covariate correlations, each MDASI item that was statistically significant in the univariate analysis was further explored simultaneously using a multivariable logistic regression model, adjusting for age, race, performance status, and cancer type, and the results were reported with the odds ratio (OR) and 95% confidence interval (95% CI).

Differential symptom analysis was done using the Mann–Whitney U test to compare the MDASI symptom item scores, MDASI interference item scores, and MDASI total scores among patients with delirium (Memorial Delirium Assessment Scale score ≥ 7) or without delirium (Memorial Delirium Assessment Scale score < 7), reporting the rank-biserial correlation (rrb) as an effect size measure for the Mann–Whitney U test. The cutoff point of 7 was selected based on established prior studies to define delirium using the Memorial Delirium Assessment Scale score [13,23]. Little’s test of missing completely at random was used to examine if missingness was completely at random. As missing data were not common, we used simple mean imputation to address any missing values in the patients who completed the MDASI assessment and thus calculate the MDASI scores. Furthermore, to examine distress as a symptom among patients with or without delirium, we used the Mann–Whitney U test to examine the MDASI score for distress as a continuous variable and the chi-square test of independence when comparing distress as a categorical variable, i.e., mild (MDASI score for distress < 5) vs. moderate-severe (MDASI score for distress ≥ 5), using cutoff thresholds from earlier research that categorized the MDASI symptoms as mild (<5 points), moderate (5–6 points), or severe (7 or higher points) [24,25,26,27].

All statistical analyses were performed using R software for Windows (R Foundation for Statistical Computing, Vienna, Austria, http://www.r-project.org, version 4.3.0, downloaded 2 January 2024). The alpha significance level was set to 0.05, reporting the two-tailed *p* value. The institutional review board of the University of Texas MD Anderson Cancer Center approved this study.

## 3. Results

### 3.1. Patient Characteristics and Presentation

Of the 243 patients included, 222 (91.4%) completed the MDASI assessment. The study cohort consisted of 243 patients with advanced cancer, with a median age of 62 years (IQR 54–69 years; Table 1). The sex distribution was nearly equal (49.4% female and 50.6% male). Most patients were White (81.1%) or Black or African American (15.6%). The most common cancer types were hematologic (20.6%), gastrointestinal (19.3%), and lung (11.9%). One or more brain lesions were present in 15.2% of patients, with 11.9% having brain metastasis and 3.3% having primary brain tumors. About half of the patients were receiving active chemotherapy (48.6%), and fewer were receiving active radiotherapy (7.8%) or other cancer treatments (3.7%).

Most patients had an acuity level of urgent (77.4%) or emergent (19.8%), with a small fraction (2.9%) being non-urgent (Table 2). The Eastern Cooperative Oncology Group performance status was predominantly 0 or 1 (62.9%), indicating a high level of functionality. The frequency of a positive delirium assessment on the Memorial Delirium Assessment Scale was 18.1% (n = 44). Common presenting symptoms included fever and nausea/vomiting (both 16.5%), shortness of breath (15.2%), and generalized weakness (8.2%).

### 3.2. Association Between MDASI Items and Mortality

**Figure 1:**
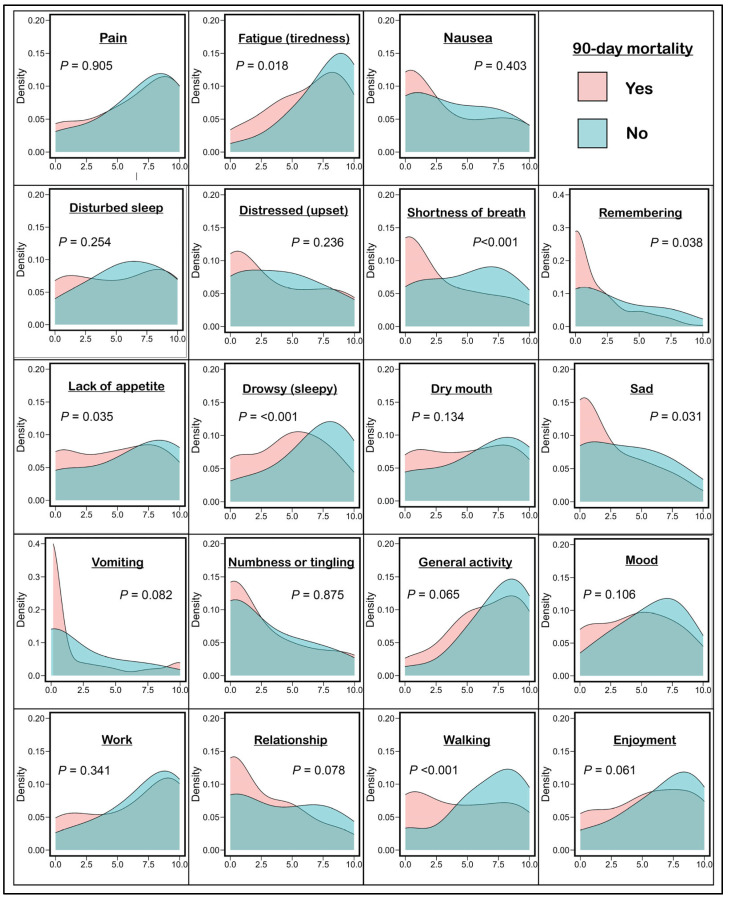
Density plots for the MD Anderson Symptom Inventory item scores stratified by 90-day mortality.

Of the MDASI symptom items, the median scores for pain and fatigue were the highest (7, IQR 4–9), and the median score for work interference was the highest (8, IQR 3–10) among the MDASI interference items (Supplementary Table S1). Most of the items (15 items) had no missing data (Supplementary Table S1). From the items with missing data, difficulty remembering and relationships with others had the highest missing values (2.3% and 1.4% respectively). The missing data was missing completely at random (Little’s test *p* = 0.055) with no identified patterns existing in the missing data. The distribution of the MDASI item scores stratified by 90-day mortality is shown in Figure 1. There was a significant difference in the MDASI score distribution in relation to 90-day mortality for fatigue (*p* = 0.018), shortness of breath (*p* < 0.001), difficulty remembering (*p* = 0.038), lack of appetite (*p* = 0.035), drowsiness (*p* < 0.001), feeling sad (*p* = 0.031), and interference with walking (*p* < 0.001; Figure 1).

In the univariate analyses, several MDASI items were associated with 90-day mortality (Table 3). These items included both MDASI symptoms (fatigue: OR 1.21, 95% CI 1.07–1.39, *p* = 0.004; shortness of breath: OR 1.17, 95% CI 1.07–1.28, *p* <0.001; difficulty remembering: OR 1.18, 95% CI 1.05–1.33, *p* = 0.007; lack of appetite: OR 1.10, 95% CI 1.01–1.21, *p* = 0.039; drowsiness: OR 1.21, 95% CI 1.08–1.36, *p* = 0.001; and feeling sad: OR 1.12, 95% CI 1.02–1.24, *p* = 0.024) and interference items (general activity: OR 1.14, 95% CI 1.01–1.30, *p* = 0.045; mood: OR 1.13, 95% CI 1.01–1.26, *p* = 0.029; and walking: OR 1.18, 95% CI 1.07–1.30, *p* < 0.001).

When adjusting for age, race, performance status, and cancer type in the multivariable analysis, only shortness of breath (adjusted OR 1.15, 95% CI 1.04–1.26, *p* = 0.005) and drowsiness (adjusted OR 1.17, 95% CI 1.05–1.33, *p* = 0.008) were associated with 90-day mortality (Table 4).

For the secondary outcomes, only difficulty remembering was associated with 14-day mortality (OR 1.30, 95% CI 1.04–1.66, *p* = 0.024), and shortness of breath with 30-day mortality (OR 1.16, 95% CI 1.02–1.33, *p* = 0.031) in the univariate analyses (Supplementary Table S2).

### 3.3. Differential Symptom Analysis in Those with or Without Delirium

**Figure 2:**
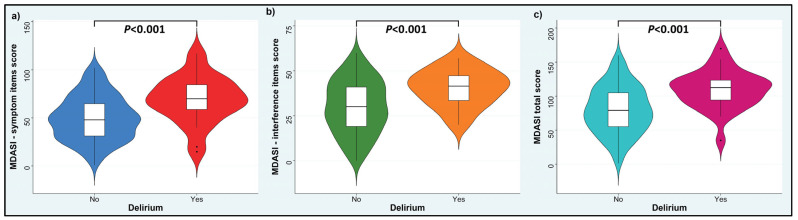
Violin plots showing the MD Anderson Symptom Inventory (MDASI) scores in patients with or without delirium. (**a**) MDASI symptom items: vomiting, shortness of breath, feeling sad, pain, numbness/tingling, nausea, lack of appetite, fatigue, dry mouth, drowsiness, disturbed sleep, feeling distressed/upset, and difficulty remembering. (**b**) MDASI interference items: mood, walking, working, general activity, relationships with others, and enjoyment of life. (**c**) Total MDASI scores.

In the differential symptom analysis, significant variances in symptom presentation were observed between the patients with delirium and those without. Patients exhibiting delirium experienced significantly more severe symptoms, based on the 13 MDASI symptom items, compared with the patients without delirium (median 70, IQR 59–85, compared with 48, IQR 31–65; *p* < 0.001; rrb = 0.45; Figure 2a). Similarly, patients with delirium had significantly more interference with daily living, based on the 6 MDASI interference items, compared with the patients who did not experience delirium (median 42, IQR 34–47, compared with 30, IQR 19–41; *p* < 0.001; rrb = 0.42; Figure 2b). The median total MDASI score was also higher in the patients who had delirium than in the patients without delirium (113, IQR 94–123, compared with 79, IQR 55–105; *p* < 0.001; rrb = 0.50; Figure 2c).

The median distress score was 3 (IQR 0–7). For the patients identified as having delirium (Memorial Delirium Assessment Scale score ≥ 7), the median distress score was significantly higher than in the non-delirium patients (5, IQR 2–8, compared with 2, IQR 0–7; *p* = 0.046; rrb = 0.23; Table 5). Similarly, moderate-severe distress was more frequent among patients with delirium than in the non-delirium patients (63.9% compared with 35.7%; *p* = 0.004; Table 5).

## 4. Discussion

Effective symptom management is essential for patients with advanced cancer presenting to the ED [28,29,30]. Early identification of warning symptoms is crucial for informing treatment choices and enhancing patient outcomes [30,31]. The current study showed that measuring symptom intensity among patients with advanced cancer in the ED setting is feasible and provides valuable information early during hospitalization, which may guide further interventions, including consultation with the palliative care service. Symptoms were notably severe, which could contribute to significant distress and poor quality of life. The MDASI scores for pain and fatigue, in particular, were higher in the current study compared with those reported previously in the general cancer patient population [17], many of whom did not have advanced stages of the disease, suggesting that symptom severity intensifies as cancer progresses. Measuring symptoms and implementing palliative care measures—including consultation with dedicated services—would potentially improve symptom control and the overall quality of life. This will support an important recommendation by the American Society of Clinical Oncology to integrate palliative care early among patients with advanced cancer [32]. Systematic symptom assessment and the development of a short, self-administered tool may help overcome some barriers complicating the integration of palliative care into the ED [33,34,35].

We found delirium to be associated with a higher symptom burden. Recognizing delirium early, even in those presenting with non-delirium related issues, helps prevent the worsening of symptoms, improving overall patient care [36,37]. In cancer patients, a multifaceted strategy is necessary to mitigate delirium in the ED, especially for those with advanced cancer. This includes early recognition, triage prioritization to identify high-risk patients, careful medication management, early supportive care, and palliative consultations, along with family support [36,37,38,39,40,41,42]. Further, difficulty remembering, as measured by the MDASI, was associated with 14-day mortality (OR 1.30, 95% CI 1.04–1.66, *p* = 0.024). Memory problems are known to be associated with delirium [43,44,45]. This finding may also be due to the disinhibition associated with delirium, and it has been shown in other studies [46,47].

Various symptoms measured by the MDASI tool, including fatigue, drowsiness, interference with walking, and interference with general activity, were associated with 90-day mortality. These symptoms may correlate with poor performance status, which has been shown to be associated with shorter survival and alteration of the quality of life [48,49,50,51]. Severe shortness of breath measured by MDASI was associated with 30-day mortality. This symptom has been associated with short survival and a negative impact on the quality of life in multiple studies [52,53,54,55]. Previously, we have shown that the presence of shortness of breath and altered mental status as presenting ED symptoms were associated with in-hospital mortality and intensive care unit admission [56].

Measuring the level of symptom distress in patients with advanced cancer can be very helpful for targeting treatment and improving their quality of life [57]. Our preliminary work showed that the presence of 2 of 3 symptoms of shortness of breath, delirium, and poor Eastern Cooperative Oncology Group performance status is associated with shorter overall survival [58]. However, as far as we know, there are no validated tools available for measuring these symptoms in the ED setting. The presence of multiple symptoms may point toward a specific diagnosis. Moreover, specific symptoms may be associated with short survival, and this will help emergency physicians focus their care towards comfort measures rather than aggressive and costly interventions.

In the ED, integrating tools like MDASI during triage or shortly after being placed in the emergency room enable the systematic assessment of symptom burden and the early detection of warning symptoms, particularly for patients at high risk, such as those with complex presentation or advanced cancer. This involves the development of a standardized workflow for identifying the high-risk patients who require symptom screening, which could involve automated alerts from the electronic health records, and the involvement of a multidisciplinary team to address any concerning symptoms, even in patients with unrelated complaints. However, barriers to this integration include potential disruptions to existing workflows in a fast-paced, high-acuity setting, the challenge of balancing intervention priorities between identified and presenting symptoms, and the difficulty of organizing timely, coordinated care from a multidisciplinary team, which should include the oncologist and the primary service.

### Limitations

Our study is limited by a relatively small number of participants and is a secondary analysis of the main study to evaluate the frequency of delirium in cancer patients presenting to the ED. Some patients did not complete the MDASI, and it remains unclear whether those who did not complete the tool experienced a higher symptom burden than those who did. Also, our study is cross-sectional, and we did not follow the participants longitudinally to show any symptom improvement after assessment in the ED. Another limitation of our study is the use of long-established data, which may not fully reflect the more recent characteristics and presentations of cancer patients, especially in the context of recent advances in novel cancer therapies such as immunotherapy and targeted therapy. These new therapies come with their own unique side effects, which could exacerbate certain symptoms like fatigue. While the findings remain relevant, further studies need to examine the applicability of our results to current clinical practice.

## 5. Conclusions

Patients with advanced cancer suffer from a high symptom burden when they present to the ED, particularly those with delirium. Specific symptoms, including shortness of breath, drowsiness, fatigue, memory difficulties, loss of appetite, sadness, and distress, are associated with poor short-term survival outcomes. Using MDASI or other symptom assessment tools to measure the symptom severity and interference with activities is feasible and provides valuable information that could help control these symptoms and improve the quality of life of the patient. The early identification of certain symptoms, including distress or cognitive impairments, can prompt targeted interventions, including psychosocial support and the consideration of palliative care. Further studies are needed to evaluate the prognostic significance of certain items in the MDASI tool.

## Figures and Tables

**Table 1:** Basic demographics and clinical characteristics of the patients with advanced cancer visiting the emergency department (n = 243).

Characteristic	No. (%)
Median age (IQR), years	62 (54–69)
Sex	
Female	120 (49.4)
Male	123 (50.6)
Race	
White	197 (81.1)
Black or African American	38 (15.6)
Others	8 (3.3)
Ethnicity	
Not Hispanic or Latino	212 (87.2)
Hispanic or Latino	29 (11.9)
Unknown	2 (0.8)
Primary cancer type	
Hematologic	50 (20.6)
Gastrointestinal	47 (19.3)
Lung	29 (11.9)
Genitourinary	26 (10.7)
Breast	25 (10.3)
Gynecologic	16 (6.6)
Others	50 (20.6)
Brain lesion(s)	
None	206 (84.8)
Brain metastasis	29 (11.9)
Primary brain tumors	8 (3.3)
Active chemotherapy	
No	125 (51.4)
Yes	118 (48.6)
Active radiotherapy	
No	224 (92.2)
Yes	19 (7.8)
Other active cancer treatment *	
No	234 (96.3)
Yes	9 (3.7)

Abbreviation: IQR, interquartile range. * Including surgery and/or hormonal therapy.

**Table 2:** Presentation of patients with advanced cancer visiting the emergency department (n = 243).

Variable	No. (%)
Acuity	
Urgent	188 (77.4)
Emergent	48 (19.8)
Non-urgent	7 (2.9)
ECOG performance status	
0	38 (15.6)
1	115 (47.3)
2	25 (10.3)
3	53 (21.8)
4	10 (4.1)
Unknown	2 (0.8)
Delirium, per Memorial Delirium Assessment Scale	
No	199 (81.9)
Yes	44 (18.1)
Delirium, per confusion assessment	
No	221 (90.9)
Yes	22 (9.1)
Presenting symptoms/signs *	
Fever	40 (16.5)
Nausea and/or vomiting	40 (16.5)
Shortness of breath	37 (15.2)
Chest pain	21 (8.6)
Cough	20 (8.2)
Generalized weakness	20 (8.2)
Back pain	16 (6.6)
Headache	13 (5.3)
Dizziness	11 (4.5)
Confusion	9 (3.7)
Extremity pain	8 (3.3)
Extremity swelling	5 (2.1)
Palpitations or arrhythmia	5 (2.1)
Fatigue	4 (1.6)
Melena or hematochezia	4 (1.6)
Abdominal pain	3 (1.2)
Constipation	3 (1.2)
Hematuria	3 (1.2)
Loss of consciousness	2 (0.8)
Fall	2 (0.8)
Abnormal lab results	2 (0.8)
Decreased appetite	2 (0.8)
Abdominal distension	1 (0.4)
Failure to thrive	1 (0.4)
Other pain sites	13 (5.3)
Other bleeding sites	5 (2.1)

Abbreviation: ECOG, Eastern Cooperative Oncology Group. * Does not add to 100% because some patients had more than one presenting symptom.

**Table 3:** Univariate analysis of the association between the MD Anderson Symptom Inventory (MDASI) items and 90-day mortality in patients with advanced cancer presenting to the emergency department (n = 243).

Variable	90-Day Mortality	
	OR (95% CI)	*p*
MDASI symptom items		
Pain	1.03 (0.94–1.14)	0.509
Fatigue	1.21 (1.07–1.39)	**0.004**
Nausea	1.04 (0.95–1.13)	0.398
Disturbed sleep	1.06 (0.97–1.16)	0.233
Feeling distressed	1.04 (0.95–1.13)	0.417
Shortness of breath	1.17 (1.07–1.28)	**<0.001**
Difficulty remembering	1.18 (1.05–1.33)	**0.007**
Lack of appetite	1.10 (1.01–1.21)	**0.039**
Drowsiness	1.21 (1.08–1.36)	**0.001**
Dry mouth	1.09 (1.00–1.20)	0.065
Feeling sad	1.12 (1.02–1.24)	**0.024**
Vomiting	1.05 (0.95–1.16)	0.297
Numbness/tingling	1.00 (0.91–1.10)	0.955
MDASI interference items		
General activity	1.14 (1.01–1.30)	**0.045**
Mood	1.13 (1.01–1.26)	**0.029**
Working	1.07 (0.98–1.18)	0.164
Relationships with others	1.09 (1.00–1.20)	0.061
Walking	1.18 (1.07–1.30)	**<0.001**
Enjoyment of life	1.10 (1.00–1.22)	0.060

Abbreviations: OR, odds ratio; CI, confidence interval. Boldface indicates *p* < 0.05.

**Table 4:** Multivariable analysis of the association between the MD Anderson Symptom Inventory items and 90-day mortality in patients with advanced cancer presenting to the emergency department (n = 243).

Variable	90-Day Mortality	
	AOR * (95% CI)	*p*
Fatigue	1.16 (1.02–1.34)	**0.031**
Shortness of breath	1.15 (1.04–1.26)	**0.005**
Difficulty remembering	1.11 (0.98–1.27)	0.098
Lack of appetite	1.07 (0.97–1.17)	0.196
Drowsiness	1.17 (1.05–1.33)	**0.008**
Feeling sad	1.08 (0.97–1.21)	0.146
Interference with general activity	1.05 (0.92–1.21)	0.490
Interference with mood	1.08 (0.97–1.22)	0.150
Interference with walking	1.09 (0.97–1.23)	0.140

Abbreviations: AOR, adjusted odds ratio; CI, confidence interval. Boldface indicates *p* < 0.05. * Adjusted for age, race, performance status, and cancer type.

**Table 5:** MD Anderson Symptom Inventory (MDASI) distress component score variations between patients with and without delirium.

Distress *	Delirium		*p*
	No	Yes	
MDASI distress score, median (IQR)	2 (0–7)	5 (2–8)	0.046
Moderate-severe distress, no. (%)			0.004
No	124 (63.9%)	10 (35.7%)	
Yes	70 (36.1%)	18 (64.3%)	

Abbreviation: IQR, interquartile range. * n = 222; 21 patients had no MDASI distress scores.

## Data Availability

The data presented in this study are available upon request from the corresponding author. The data are not publicly available, and an IRB approval from the MD Anderson Cancer Center is required to share the data.

## Supplementary Materials

The following supporting information can be downloaded at https://www.mdpi.com/article/10.3390/cancers16233988/s1, Table S1: Median MD Anderson Symptom Inventory (MDASI) scores in patients with advanced cancer presenting to the emergency department (n = 243). Table S2: Univariate analysis of the association between MD Anderson Symptom Inventory items and 14- or 30-day mortality in patients with advanced cancer presenting to the emergency department (n = 243). Supplemental methods: Sample size calculations.
