# Supplementary material for: Severity of Symptoms as an Independent Predictor of Poor Outcomes in Patients with Advanced Cancer Presenting to the Emergency Department: Secondary Analysis of a Prospective Randomized Study
Source: Cancers (Basel). 2024 Nov 28;16(23):3988. doi: 10.3390/cancers16233988 (PMC11640218; doi:10.3390/cancers16233988)
Supplement: Supplementary file 1 [file cancers-16-03988-s001.zip › cancers-3303248-supplementary.pdf]

SUPPLEMENTAL DATA

SUPPLEMENTAL TABLES

Table S1. Median MD Anderson Symptom Inventory (MDASI) scores in patients with advanced cancer presenting to the emergency department (n=222).

Variable	Median (IQR)	Missing, %
MDASI symptom items		
Pain	7 (4–9)	0.0
Fatigue	7 (4–9)	0.0
Nausea	2 (0–7)	0.0
Disturbed sleep	5 (2–8)	0.0
Feeling distressed	3 (0–7)	0.0
Shortness of breath	2 (0–6)	0.0
Difficulty remembering	0 (0–3)	2.3
Lack of appetite	5 (0–8)	0.0
Drowsiness	5 (3–8)	0.0
Dry mouth	5 (2–8)	0.0
Feeling sad	2 (0–5)	0.0
Vomiting	0 (0–2)	0.0
Numbness/tingling	0 (0–5)	0.5
MDASI interference items		
General activity	7 (5–9)	0.5
Mood	5 (2–8)	0.0
Working	8 (3–10)	1.4
Relationships with others	2 (0–5)	0.0
Walking	5 (0–8)	0.0
Enjoyment of life	6 (3–9)	0.0

Table S2. Univariate analysis of the association between MD Anderson Symptom Inventory items and 14- or 30-day mortality in patients with advanced cancer presenting to the emergency department (n=243).

	14-day mortality		30-day mortality	
**Variable**	**OR (95% CI)**	***P***	**OR (95% CI)**	***P***
Pain	0.91 (0.75-1.12)	0.349	1.04 (0.90-1.22)	0.613
Fatigue	1.14 (0.87-1.59)	0.389	1.11 (0.92-1.37)	0.304
Nausea	1.03 (0.85-1.25)	0.729	1.02 (0.89-1.17)	0.750
Disturbed sleep	0.97 (0.79-1.18)	0.731	0.99 (0.86-1.14)	0.893
Feeling distressed	0.97 (0.79-1.17)	0.753	1.05 (0.92-1.20)	0.502
Shortness of breath	1.12 (0.93-1.37)	0.231	1.16 (1.02-1.33)	**0.031**
Difficulty remembering	1.30 (1.04-1.66)	**0.024**	1.11 (0.93-1.31)	0.241
Lack of appetite	1.01 (0.84-1.22)	0.953	1.05 (0.92-1.21)	0.465
Drowsiness	1.30 (1.02-1.81)	0.065	1.14 (0.98-1.36)	0.107
Dry mouth	1.25 (0.98-1.72)	0.100	1.06 (0.92-1.24)	0.418
Feeling sad	1.00 (0.79-1.24)	0.977	1.00 (0.85-1.16)	0.950
Vomiting	1.03 (0.84-1.24)	0.730	1.04 (0.90-1.19)	0.568
Numbness/tingling	0.92 (0.71-1.12)	0.449	0.91 (0.77-1.05)	0.236
General activity	1.02 (0.77-1.44)	0.877	1.03 (0.85-1.28)	0.775
Mood	1.03 (0.83-1.30)	0.775	1.02 (0.88-1.20)	0.781
Working	1.12 (0.87-1.57)	0.447	1.01 (0.86-1.21)	0.864
Relationships with others	1.09 (0.89-1.34)	0.396	1.13 (0.98-1.30)	0.085
Walking	1.00 (0.77-1.29)	0.973	1.07 (0.90-1.27)	0.449
Enjoyment of life	0.92 (0.73-1.18)	0.512	1.06 (0.90-1.25)	0.506

Abbreviations: OR, odds ratio; CI, confidence interval
Boldface indicates *P*<0.05

SUPPLEMENTAL METHODS

Sample size calculations: This is a single-arm observational study that determined the prevalence of delirium among patients with advanced cancer who presented to MD Anderson Emergency Department. The primary endpoint was the proportion of patients with delirium, as defined by the Confusion Assessment Method instrument, among all patients who meet the entry criteria for the study. All patients meeting the entry criteria during the enrollment period were enrolled until the sample size requirement has been met. Sample size was calculated under the assumption that with a sample size of 500 patients, a 95% confidence interval around the proportion with delirium will be no wider than +/- 4.4%. An interim look for futility will be conducted after the first 100 patients have been enrolled and observed. With 100 patients, if the true delirium rate is 10% we will have a 93.89% likelihood of observing at least 6 delirious patients. Accrual will be suspended and a sample size re-adjustment considered if we observe fewer than 6 delirious patients in the first 100 enrolled.
